# The application of high-throughput sequencing technology to analysis of *amoA* phylogeny and environmental niche specialisation of terrestrial bacterial ammonia-oxidisers

**DOI:** 10.1186/s40793-019-0342-6

**Published:** 2019-07-04

**Authors:** Axel Aigle, James I. Prosser, Cécile Gubry-Rangin

**Affiliations:** School of Biological Sciences, Cruickshank Building, University of Aberdeen, St. Machar Drive, Aberdeen, AB24 3UU UK

**Keywords:** Nitrification, *amoA*, 16S rRNA, Archaea, Bacteria, Illumina MiSeq, pH

## Abstract

**Background:**

Characterisation of microbial communities increasingly involves use of high throughput sequencing methods (e.g. MiSeq Illumina) that amplify relatively short sequences of 16S rRNA or functional genes, the latter including ammonia monooxygenase subunit A (*amoA)*, a key functional gene for ammonia oxidising bacteria (AOB) and archaea (AOA). The availability of these techniques, in combination with developments in phylogenetic methodology, provides the potential for better analysis of microbial niche specialisation. This study aimed to develop an approach for sequencing of bacterial and archaeal *amoA* genes amplified from soil using bioinformatics pipelines developed for general analysis of functional genes and employed sequence data to reassess phylogeny and niche specialisation in terrestrial bacterial ammonia oxidisers.

**Results:**

*amoA* richness and community composition differed with bioinformatics approaches used but analysis of MiSeq sequences was reliable for both archaeal and bacterial *amoA* genes and was used for subsequent assessment of potential niche specialisation of soil bacteria ammonia oxidisers. Prior to ecological analysis, phylogenetic analysis of *Nitrosospira*, which dominates soil AOB, was revisited using a phylogenetic analysis of 16S rRNA and *amoA* genes in available AOB genomes. This analysis supported congruence between phylogenies of the two genes and increased previous phylogenetic resolution, providing support for additional gene clusters of potential ecological significance. Analysis of environmental sequences using these new sequencing, bioinformatics and phylogenetic approaches demonstrated, for the first time, similar niche specialisation in AOB to that in AOA, indicating pH as a key ecological factor controlling the composition of soil ammonia oxidiser communities.

**Conclusions:**

This study presents the first bioinformatics pipeline for optimal analysis of Illumina MiSeq sequencing of a functional gene and is adaptable to any amplicon size (even genes larger than 500 bp). The pipeline was used to provide an up-to-date phylogenetic analysis of terrestrial betaproteobacterial *amoA* genes and to demonstrate the importance of soil pH for their niche specialisation and is broadly applicable to other ecosystems and diverse microbiomes.

**Electronic supplementary material:**

The online version of this article (10.1186/s40793-019-0342-6) contains supplementary material, which is available to authorized users.

## Background

Despite advances in our understanding of niche specialisation of ammonia oxidisers during the past 20 years, the successive discoveries of archaeal ammonia oxidisers (AOA) [1] and complete ammonia oxidisers (comammox) [2, 3] have focussed recent research activities on these organisms. In particular, in terrestrial environments, pH has been described as the most important soil factor regulating the AOA ecological and evolutionary adaptation [4, 5] and, while there is currently insufficient information on which to assess comammox growth or adaptation in this environment, the ecophysiology of soil comammox appears to differ from that of strains that have been cultivated and described to date [6]. In contrast, the modern technological and methodological advances have not benefited analysis of the longer-known bacterial ammonia oxidisers (AOB), despite demonstration of their significant role in nitrification, particularly in managed and heavily fertilised agricultural soils [7–9]. In particular, phylogenetic analyses have gained in complexity and consequent accuracy, and finer phylogenetic analyses of soil AOB [10–14] would benefit from increases in knowledge of the diversity of soil AOB since previous in-depth studies (e.g. [15]). In particular, reassessment of the terrestrial *Nitrosospira* phylogeny is required using more advanced phylogenetic methods, as this genus dominates soil AOB communities.

Increased phylogenetic resolution improves the ability to assess microbial niche specialisation, at least in organisms whose phylogenetic structure reflects their ecological niche [16]. Several environmental factors have been proposed as important for controlling the community structure of terrestrial AOB, including the nature and quantity of organic matter [17], mean annual temperature [18], amount of nitrogen fertilisation [19] and soil pH [20]. However, understanding of the niche differentiation of these microorganisms in natural unfertilised environments remains limited and the role of terrestrial *Nitrosospira* in nitrous oxide emissions [8, 9] justifies the need for an improved analysis of their niche specialisation to clarify their ecological adaptation and environmental impact. This analysis would specifically test the extent to which pH is a key factor controlling the ecological distribution of AOB in soils, as previously demonstrated for AOA in natural environments [4, 5], by using a soil pH gradient of several unfertilised land management (grassland, forest, agricultural or moorland) soils.

Many studies target the 16S rRNA gene to characterise phylogenetic diversity within a sample, while others target genes involved in specific ecosystem functions, facilitating ecological and evolutionary predictions within a functional group. The ammonia monooxygenase subunit A (*amoA*) gene targets an ecosystem function, ammonia oxidation, which is the rate-limiting step in nitrification, a key process in biogeochemical cycling of nitrogen. The *amoA* gene has been extensively used to estimate the abundance and diversity of bacterial (AOB) and archaeal ammonia oxidisers (AOA) and has provided evidence for their high phylogenetic diversity in natural environments, including soil [21]. Therefore, the *amoA* gene was chosen as the optimal functional gene to analyse soil AOB diversity and two sets of primers have been mainly employed in terrestrial environments, amoA-1F/amoA-2R [22] and CrenamoA23f/CrenamoA616r [23], amplifying 429-bp and 629-bp fragments of bacterial and archaeal *amoA* genes, respectively. Developments in high-throughput sequencing (HTS) technologies have greatly increased our ability to characterise natural microbial communities, through significant increases in the depth and accuracy of sequencing of genes of interest, amplified from environmental DNA (see [24] for a recent review). Currently, one of the most commonly applied approaches is short-read sequencing (e.g. Illumina) technology, producing a high number of sequences (> 15 Gbp per Illumina MiSeq V3 run) with high accuracy (99.9% at QC30) but short sequence length (< 500 bp). Surprisingly, this approach has not been applied to terrestrial ammonia oxidisers using the above primers [21, 22], probably due to the large size of the amplicons (mainly for the AOA). Therefore, this study provided the opportunity to develop a bioinformatics pipeline for amplicons of various size (including those > 500-bp) and its validity was assessed by comparison with a previously acquired 454 sequencing dataset with known phylogenetic resolution [4].

The aims of this study were, therefore, i) to revisit the *amoA* phylogeny of terrestrial *Nitrosospira* and improve its phylogenetic resolution; ii) to improve the analysis of niche specialisation of terrestrial *Nitrosospira* to clarify their ecological adaptation and test the extent to which pH is a key factor in their ecological distribution; and iii) to provide an Illumina MiSeq V3 sequencing approach for environmental analysis of the ammonia monooxygenase subunit A (*amoA*) gene present in both bacterial and archaeal ammonia oxidisers, applicable to other functional genes.

## Results and Discussion

The aims of the study were addressed by performing archaeal and bacterial *amoA* gene Illumina MiSeq sequencing, respectively, on 47 and 33 UK soils for which environmental data were available. In addition, 16S rRNA and *amoA* genes retrieved from 56 available *Nitrosospira* strains were used for phylogenetic reconstructions and assessment of phylogenetic congruency between them.

### *Nitrosospira* classification

**Fig. 1:**
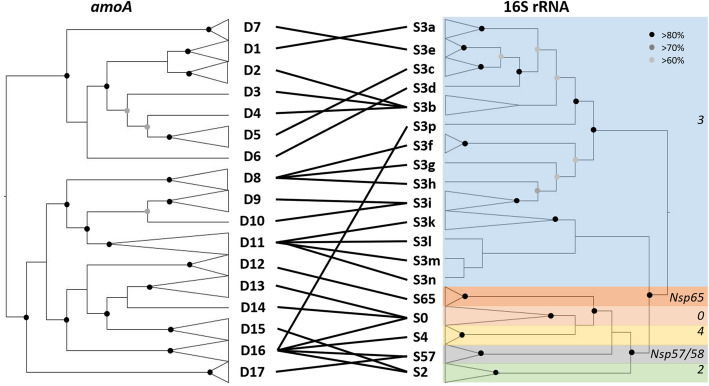
Congruence between *amoA* and 16S rRNA gene cladograms for *Nitrosospira* cultures and genomes used in this study. Lines represent correspondence between clusters of the two phylogenies. Names of the phylogenetic clusters indicated in bold on the two phylogenetic trees are arbitrary, while those in italics correspond to previously published 16S rRNA phylogenies [28, 29]. Details of the strain identity within each cluster are given in Table 1 and node bootstrap values are indicated by circles with different shadings

Two genera of betaproteobacterial ammonia oxidisers have been described, *Nitrosomonas* and *Nitrosospira* [25], the latter including two previously described genera, *Nitrosolobus* [26] and *Nitrosovibrio* [27]. This study focused on the *Nitrosospira* genus, which dominates betaproteobacterial ammonia oxidiser communities in soil. The 16S rRNA gene sequences from cultivated *Nitrosospira* isolates and those amplified from environmental samples have previously been classified within seven lineages, for which phylogenetic node support was not high [10–14]. Sequences of six of these lineages were retrieved in the present study at a high taxonomic ranking (sub-clades) using 56 available *Nitrosospira* strains (Fig. 1 and Additional file 1: Figure S1), the exception being cluster 1 [10], for which no cultured isolate has yet been obtained. Analysis of *amoA* and 16S rRNA gene sequences of these *Nitrosospira* strains, employing a Maximum-Likelihood phylogenetic framework, delineated 17 and 19 phylogenetic clusters, respectively (Fig. 1 and Additional file 1: Figure S1, Table 1), with most of the diversity being within the previously defined cluster 3. The majority of the 56 AOB strains analysed here contained a single *amoA* gene copy (Additional file 1: Table S1), but several *Nitrosospira* spp. genomes contain > 1 different but highly homologous *amoA* genes, which probably originated from duplication events rather than horizontal gene transfer [30–32]. This finding, along with the presence of > 1 *amoA* gene copy in most described *Nitrosomonas* genomes [33], has important consequences for quantification of terrestrial AOB in environmental communities using quantitative PCR analysis of *amoA* genes. Most nodes at the roots of individual clusters in both phylogenetic trees were strongly supported (> 80%), while support for more ancestral phylogenetic branching was supported for most of the nodes in both the 16S rRNA gene and the *amoA* gene phylogenies, even if some paraphyletic branching could not be resolved (Fig. 1 and Additional file 1: Figure S1).

**Table 1:** Terrestrial *Nitrosospira* AOB strains used in this study with related *amoA* and 16S rRNA gene phylogenetic affiliations (based on the phylogenetic trees presented in Fig. 1)

Organism	*amoA* gene lineage	16S rRNA gene lineage
*Nitrosospira multiformis* Nsp16	D1	S3a
*Nitrosospira sp.* Nsp18	D1	S3a
*Nitrosospira sp.* 1 Nsp11	D1	S3a
*Nitrosospira briensis* C-128	D2	S3b
*Nitrosospira sp.* Nsp1	D2	S3b
*Nitrosospira sp.* NRS527	D2	S3b
*Nitrosospira sp.* Nsp40	D3	S3b
*Nitrosospira sp.* Nsp22	D4	S3b
*Nitrosospira tenuis* Nv12	D5	S3c
*Nitrosospira sp.* Nv6	D5	S3c
*Nitrosospira sp.* Nsp37	D5	S3c
*Nitrosospira sp.* Nv4	D5	S3c
*Nitrosospira sp.* Nsp62	D6	S3d
*Nitrosospira briensis* Nsp10	D7	S3e
*Nitrosospira briensis* Nsp8	D7	S3e
*Nitrosospira sp.* Nsp14	D8	S3f
*Nitrosospira sp.* Nsp17	D8	S3f
*Nitrosospira sp.* Nsp2	D8	S3 g
*Nitrosospira sp.* Nsp44	D8	S3 h
*Nitrosospira multiformis* 24C	D9	S3i
*Nitrosospira sp.* L115	D9	S3i
*Nitrosospira sp.* A16	D9	S3i
*Nitrosospira sp.* AF	D9	S3i
*Nitrosospira tenuis* Nv1	D10	S3i
*Nitrosospira multiformis* ATCC 25196	D11	S3k
*Nitrosospira multiformis* Nl13	D11	S3k
*Nitrosospira multiformis* Nl4	D11	S3k
*Nitrosospira multiformis* Nl18	D11	S3k
*Nitrosospira multiformis* Nl15	D11	S3k
*Nitrosospira multiformis* Nl14	D11	S3k
*Nitrosospira multiformis* Nl7	D11	S3k
*Nitrosospira multiformis* Nl8	D11	S3k
*Nitrosospira multiformis* Nl12	D11	S3 l
*Nitrosospira multiformis* Nl2	D11	S3 m
*Nitrosospira multiformis* Nl3	D11	S3n
*Nitrosospira sp.* Nsp65	D12	S65
*Nitrosospira sp.* 56–18	D12	S65
*Nitrosospira sp.* Nsp5	D13	S0
*Nitrosospira multiformis* Nl1	D13	S0
*Nitrosospira sp.* Nsp13	D13	S0
*Nitrosospira sp.* Nsp6	D13	S0
*Nitrosospira sp.* Nsp12	D13	S0
*Nitrosospira sp.* 40KI	D13	S0
*Nitrosospira sp.* NpAV	D14	S0
*Nitrosospira lacus* APG3	D16	S0
*Nitrosospira sp.* Ka4	D16	S4
*Nitrosospira sp.* Ka3	D16	S4
*Nitrosospira sp.* Nsp41	D16	S3p
*Nitrosospira sp.* Nsp58	D16	S57
*Nitrosospira sp.* B6	D16	S2
*Nitrosospira sp.* III7	D15	S2
*Nitrosospira sp.* O13	D15	S2
*Nitrosospira sp.* O4	D15	S2
*Nitrosospira sp.* AHB1	D15	S2
*Nitrosospira sp.* Nsp57	D17	S57
*Nitrosospira sp.* Nl5	D17	S57

The present phylogenetic approach demonstrates that the previous 16S rRNA gene phylogenetic clustering for lineage 3 requires future refinement, as this previously described cluster is paraphyletic and contains numerous distinct clusters. While the present phylogenetic analysis did not aim to represent the extent of *Nitrosospira* diversity (on neither 16S rRNA or *amoA* genes), it provides evidence for the existence of clear and distinct lineages using both marker genes of cultivated strains (Fig. 1; Table 1 and Additional file 1: Table S1) and, in contrast to earlier analysis [34], demonstrated a strong phylogenetic congruence between *amoA* and 16 rRNA genes using a tanglegram approach (Fig. 1 and Additional file 1: Figure S2).

### Niche specialisation of terrestrial bacterial ammonia oxidisers

**Fig. 2:**
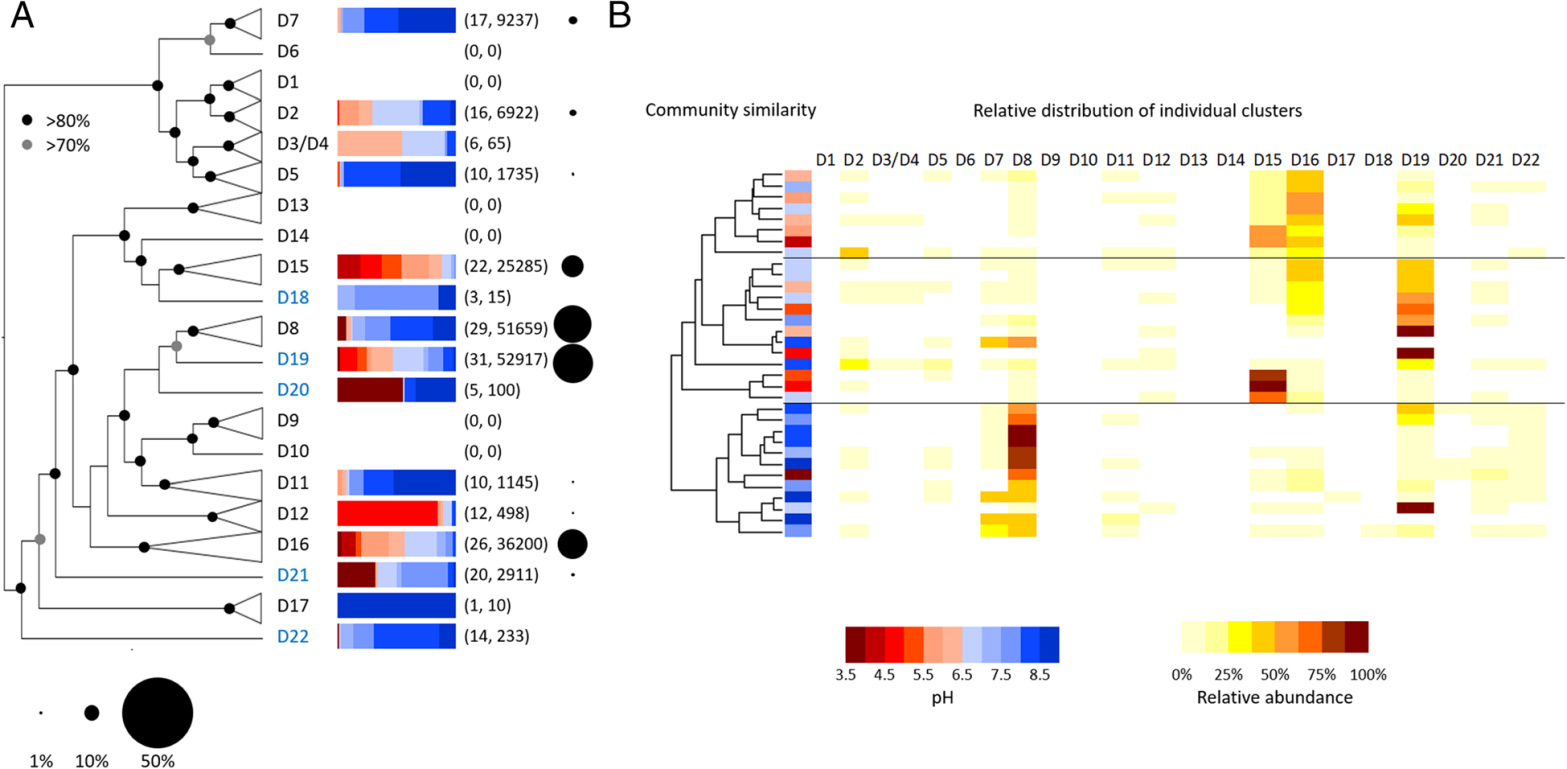
**a** Cladogram representing bacterial *amoA Nitrosospira* diversity in 33 UK soils based on Illumina MiSeq sequences. Names in blue indicate the absence of an *amoA* gene sequence from a cultivated *Nitrosospira* strain or representative genome. The bars and circles represent the distribution of sequences within each phylogenetic cluster and their relative abundance, respectively. Numbers in brackets indicate the number of sites and sequences in each phylogenetic cluster, respectively. **b** Dendrogram representing the relatedness of *Nitrosospira* bacterial ammonia oxidiser communities in 33 UK soils (pH 3.5–8.7) based on the relative abundance of distinct lineages of bacterial *amoA* genes. The relative abundance of each phylogenetic lineage in each soil is displayed as a heat map. The soil pH from each site is indicated by red-blue panels, in the range 3.5–9 at intervals of 0.5. The relative abundance of sequences within an individual cluster is indicated by yellow-brown shading at 12.5% intervals

A large majority (99.98% of the 199,295 sequences) of the environmental sequences obtained from the 33 soils amplified using bacterial primers affiliated to the *Nitrosospira* genus, which has been described as the most abundant AOB genus in unfertilised soils, while *Nitrosomonas* phylotypes are more frequently retrieved from fertilised or ammonia-rich environments [30]. These *amoA* sequences were affiliated to ecologically coherent phylogenetic AOB clusters (Fig. 2a) and multivariate statistical analysis of their ecological distribution demonstrated greatest correlations with soil pH and, to a lesser extent, C:N ratio across a range of phylogenetic scales (Table 2, see Additional file 1: Table S2 for detailed results). There is strong evidence for pH specialisation of AOA [4], but this strong influence of pH on AOB phylotype distribution over multiple phylogenetic scales has not been described previously, suggesting a role for pH in determining community composition of all terrestrial ammonia oxidisers. A heatmap representing the relative abundance of each phylogenetic cluster in each soil indicated higher relative abundance of four clusters (D8, D15, D16 and D19) (Fig. 2b) and dominance of these clusters strongly influenced the general pH association with overall community similarity pattern. Sequences affiliating to the cluster D8 were particularly abundant in neutro-alkaline soils. In contrast to such classical AOB ecological niche distribution, most AOB sequences in neutro-acidic soils affiliated with the D15, D16 and D19 clusters, but more genomes and cultures representative of these clusters are required for characterisation of their phenotypes. For example, AOB ureolytic activity has been proposed, among others, as a growth strategy in acidic conditions [21]. Other less abundant phylogenetic clusters would also benefit from further genomic and cultivation efforts (see Fig. 2a).

**Table 2:** Correlations between measured environmental factors and relative abundance of bacterial ammonia oxidiser lineages (identified at different identity thresholds) in 33 soils used for analysis of bacterial *amoA* gene sequences

Environmental factors	Identity (%)			
	90	95	97	100
pH	0.001***	0.001***	0.001***	0.001***
C	0.274	0.003**	0.006**	0.001***
N	0.166	0.016*	0.020*	0.001***
C:N	0.001***	0.002**	0.003**	0.002**
Moisture	0.459	0.264	0.112	0.015*
LOI	0.139	0.198	0.032*	0.072.
Vegetation	0.406	0.172	0.083 .	0.106
Number of clusters	14	71	187	3868

Significance codes: *p* < 0.001 ***; *p* < 0.01 **; *p* < 0.05 *

### Comparison of sequencing technologies

**Fig. 3:**
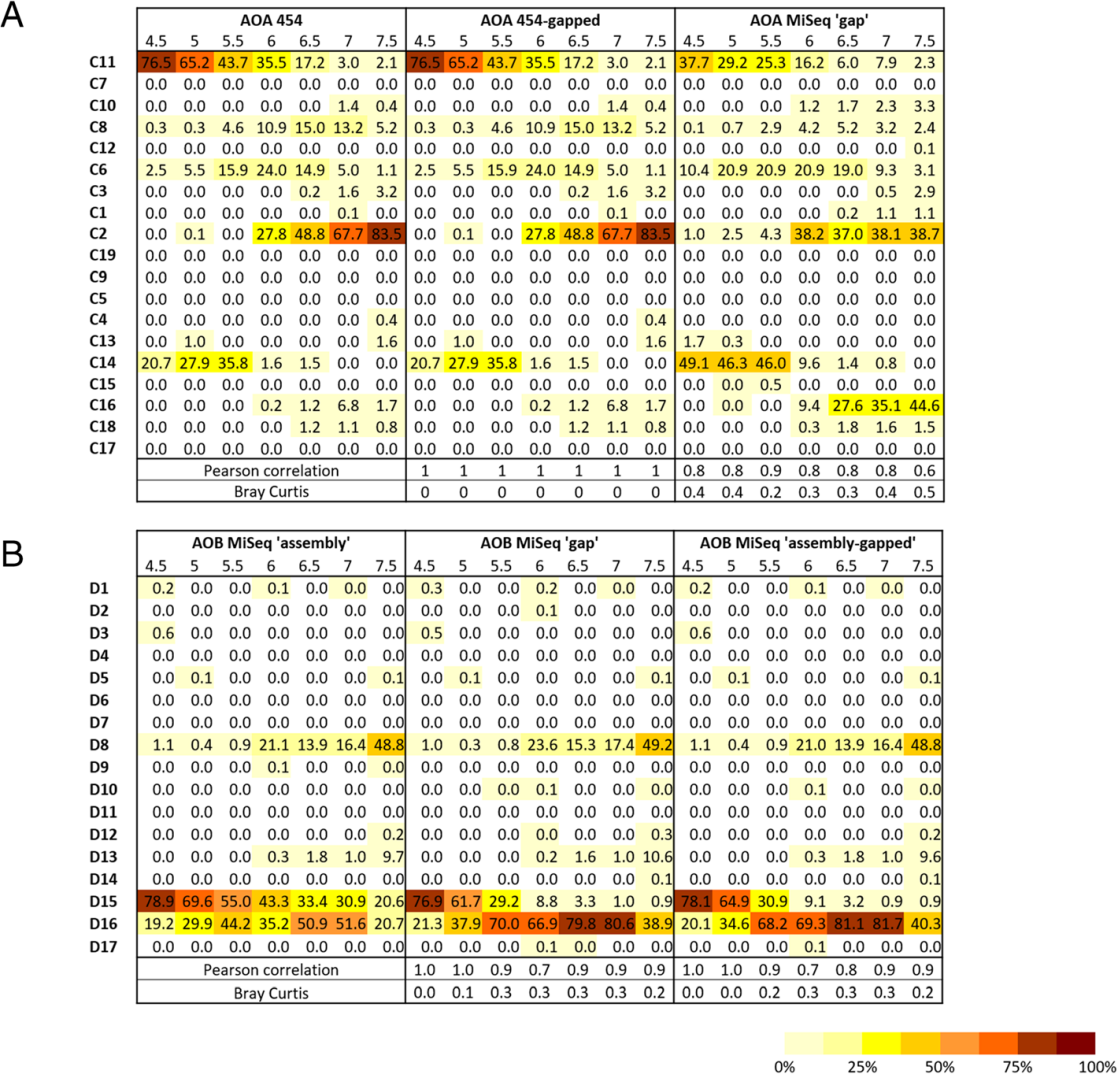
Heatmap representing the relative abundance of archaeal (**a**) and bacterial (**b**) *amoA* gene sequences within each phylogenetic cluster, produced using different sequencing technologies. The row below each heatmap indicates the Pearson and Bray-Curtis correlations of the microbial community structure for each soil (pH 4.5–7.5) relative to the technology considered the most accurate

Comparisons of the diversity of environmental *amoA* gene sequences were made using both OTU richness (at 100% sequence similarity) and microbial community composition for archaea and bacteria independently using the different sequencing approaches, 454 (only available for AOA and previously described in [4]) and Illumina MiSeq sequencing. Both measures varied with sequencing technology and associated bioinformatics pipeline (Fig. 3; Additional file 1: Table S3). Despite a low error rate for Illumina sequencing, the read length (2 × 300 bp) limited the number of cleaned sequences (Additional file 1: Table S3). Two bioinformatics pipelines (‘assembly’ vs. ‘gap’) were used to analyse the AOB *amoA* amplicons (490-bp), due to overlapping of the paired-end sequences. Rarefied richness was similar or higher for the ‘gap’ pipeline than the ‘assembly’ pipeline, due to the higher restrictive size selection in the latter (Additional file 1: Table S3). Despite detection of similar to greater richness, phylogenetic assignment of sequences was differentially affected by the pipeline used (Bray-Curtis ≥0.3 for 4 soils; Fig. 3b). Community dissimilarity obtained using the full-length ‘assembly’ and ‘assembly-gapped’ sequences indicated that the ‘gapped’ region contains important phylogenetic information, especially for the sequences present in acido-neutral soils (Fig. 3b). However, a similar comparative analysis of AOA *amoA* sequences, using previously obtained 454 sequences (i.e. 454 full-length sequences vs 454-gapped sequences), indicated that deletion of the central archaeal *amoA* gene region did not impact significantly on estimated archaeal community composition (Bray-Curtis = 0 for all 7 soils; Fig. 3a) or phylogenetic reconstruction (Additional file 1: Figure S4; Euclidian distance between the 2 trees = 0.28). The difference in community composition between the AOA 454 and the AOA MiSeq ‘gap’ (Fig. 3a) certainly derives from different sampling dates for the 2 different technologies. Therefore, these analyses were used to validate the MiSeq ‘gap’ and ‘assembly’ bioinformatics pipelines for AOA and AOB *amoA* sequencing, respectively). These findings suggest that Illumina MiSeq sequencing can be successfully used to provide a good characterisation of AOA and AOB *amoA* amplicon sequences to infer their community structure. The sequencing bioinformatics pipelines presented here are freely available on GitHub (https://github.com/AigleAxel/amoA_MiSeq_sequencing/) allowing their implementation for other functional genes of interest. Specific advantages and associated limitations of each bioinformatics pipeline (e.g. high recovered diversity for the ‘gap’ pipeline and high confidence of sequence phylogenetic affiliation for the ‘assembly’ pipeline) indicate the requirement for thorough comparison of approaches for cleaning of sequencing data for any novel analysed gene.

## Conclusions

This study offers an optimal bioinformatics pipeline for high-throughput sequencing of functional genes, adaptable to any amplicon size and freely available on GitHub (https://github.com/AigleAxel/amoA_MiSeq_sequencing/). This tool will be useful to the researchers interested in diverse microbiome communities, especially those working on functional genes of interest larger than 500 bp. The developed pipeline was used to provide a revisited and up-to-date phylogenetic analysis of terrestrial betaproteobacterial ammonia oxidiser *amoA* genes and further analysis provided evidence for soil pH as a key ecological factor controlling the niche specialisation of those microbes.

## Material and methods

### *Sample origin, DNA extraction and* amoA *amplification*

Bacterial and archaeal *amoA* sequences were obtained from 26 and 39 soils, respectively, selected from the UK Countryside Survey (http://www.countrysidesurvey.org.uk/) and from 7 soil samples from long-term experimental field plots maintained for more than 60 years at a range of soil pH values (SRUC, Craibstone, Scotland, grid reference NJ872104). Soils were selected to include a wide range of characteristics (Additional file 1: Table S4), including pH (3.48–8.55), C:N ratio (8.5–22.1) and moisture content (14.2–75.1%), across several ecosystem managements (agricultural, forest, grassland, moorland), and a previous study [4] suggests that they contain the full range of currently known mesophilic terrestrial AOA.

Soil DNA was extracted as described by [35] and modified by [36] and bacterial and archaeal *amoA* genes were amplified, respectively, using primers amoA-1F/amoA-2R [22] and CrenamoA23f/CrenamoA616r [23] containing additional specific MiSeq-tailed sequences, following manufacturers’ recommendations. All amplifications were performed in a 25-μl reaction using the KAPA HiFi HotStart ReadyMix (Kapa Biosystems) with 0.4 μM of each primer and 40–60 ng of template. Thermal cycling conditions were 95 °C for 3 min followed by 35 cycles of 98 °C for 20 s, 58 °C for 15 s, 72 °C for 15 s or 20 s for bacterial and archaeal *amoA* respectively, followed by 72 °C for 5 min. Bacterial *amoA* MiSeq sequencing was performed on all 33 soil samples, allowing the construction of a non-redundant database of representative soil AOB *amoA* sequences to complement an equivalent database of AOA *amoA* sequences obtained using 454 sequencing on 46 soils [4].

### High throughput sequencing

Prior to MiSeq Illumina sequencing, PCR-amplified sequences were cleaned using AMPure® XP beads (Beckman Coulter) and PCR-indexing was performed using the Nextera XT Index Kit according to the manufacturer’s protocol. Following further cleaning, library quantification, normalisation and pooling of 144 samples per sequencing run were performed prior to paired-end V3 MiSeq sequencing, enabling production of 2 × 300-bp reads.

### Sequence analysis bioinformatic pipelines

The different read lengths of archaeal and bacterial *amoA* genes generated by Illumina MiSeq sequencing necessitated use of two read assembly strategies. The first, with overlapping reads and termed the ‘assembly’ pipeline, was used for bacterial *amoA* only. The second, with non-overlapping reads and termed the ‘gap’ pipeline, was used for both archaeal and bacterial *amoA*. For the ‘assembly’ pipeline, reads were demultiplexed using NextGENe software and, for each sample, paired-end raw reads were trimmed (−- paired) with very low quality filtration (−q 15) using Trim Galore (V0.4.5, [37]) and further filtered using the filterAndTrim command from the DADA2 package (maxEE = c(2,2), maxN = 0) [38]. Minimal size selection and read truncation (truncLen = c(229,229), minLen = 229) were applied to allow overlap of 10 bases between the paired-end reads. Assembly was performed using the paired-end assembler PEAR with default parameters [39] and assembled reads were size-selected using usearch (−fastx_truncate -trunclen 448) [40] and subsequently dereplicated at 100% sequence identity (while keeping the read abundance information) using usearch (−cluster_fast -centroids -sizeout). Finally, dereplicated reads were translated and any read that included a stop codon was deleted prior to removal of chimera and singletons using unoise3 [41].

Initial steps of the ‘gap’ pipeline were similar to the ‘assembly’ pipeline except that minimal size selection was modified (−length 200), as a compromise between selection of high-quality reads (especially for reverse reads) and conservation of high-quality information (carried by higher number of nucleotides) to maximise the output number of reads. The reverse reads were reverse-complemented and concatenated with the forward reads (instead of being assembled via PEAR). The following steps (dereplication, amino acid translation, chimera and singleton removal) were the same as in the ‘assembly’ pipeline.

Comparison of the different sequencing methodologies was performed for the seven Craibstone samples following a blast-assignment of sequences to the different phylogenetic clusters (see [5] and below for AOA and AOB *amoA* databases, respectively) and the proportions of sequences affiliated to each phylogenetic cluster within each soil sample were represented in a heatmap. As the two different bioinformatics pipelines produced AOB sequences of different lengths, the community dissimilarity between full-length ‘assembly’ and ‘assembly-gapped’ sequences (i.e. ‘assembly’ sequences for which the corresponding gap region of the ‘gap’ pipeline was deleted) were tested on the AOB Illumina MiSeq dataset. Similarly, the significance of the deleted sequence produced in the ‘gap’ AOA Illumina MiSeq dataset was tested using previously obtained 454 sequences (i.e. 454 full-length sequences vs 454 sequences without the DNA region corresponding to the MiSeq ‘gap’ and termed ‘454-gapped’). Pearson correlation and Bray-Curtis similarity indices were estimated for each soil sample independently using the cor() and the vegdist() functions from the vegan package on R V3.5.1 [42, 43], respectively, by using the communities produced using the longest read assembly as reference (AOB MiSeq ‘assembly’ and AOA 454 for AOB and AOA, respectively). Finally, OTU richness of each dataset was estimated using the rarefy function from the vegan package [43] in R, with or without rarefaction to the smallest number of sequences obtained in the different sequencing approaches. The technologies were also compared by phylogenetic comparison using the 454-gapped archaeal *amoA* sequences (see details of phylogenetic reconstruction below) by estimating the Euclidian distance between the two *amoA* trees (built with either full- or truncated-length) using treecompare in the DendroPy library [44].

### Phylogenetic tree analysis

Known terrestrial AOB are affiliated to the *Nitrosospira* genus (including the previously named *Nitrosolobus* and *Nitrosovibrio* strains). The *amoA* and 16S rRNA gene sequences of 56 *Nitrosospira* strains were recovered from NCBI and JGI databases (see Additional file 1: Table S1), were considered as reference sequences and were used to build reference Maximum-Likelihood phylogenetic trees (see below). The AOB *amoA* reference sequences were also merged with the dereplicated AOB *amoA* MiSeq sequences (from the ‘assembly’ pipeline) and another phylogenetic tree was constructed to assess global diversity in soil. Finally, the dereplicated AOA *amoA* sequences previously produced on 46 UK soils using 454 were trimmed in the central sequence region (corresponding to the gap region of the MiSeq ‘gap’ pipeline and resulting in 264 bp) and a Bayesian phylogenetic tree was constructed to compare to a previously published phylogenetic tree [5].

All sequence datasets were aligned using Mafft [45] and further processed with TrimAl [46] with “- gappyout” flag. For all *amoA* datasets, any sequence in which a recombination event was detected using at least 3 of 4 methods (RDP, Bootscan, GENECOV and MaxChi) implemented in RDP4 software [47] was removed after manual curation. Codon saturation was detected by comparing the maximum likelihood distance and the number of differences (Pairwise distance, MEGA 6.06, [48]) for each codon between each pair of sequences. This was statistically assessed using the Xia test implemented in DAMBE [49] and the third codon position was removed.

All AOB phylogenetic trees (both *amoA* and 16S rRNA trees) were constructed using IQ-TREE [50] (with partitioning of the 2 codon positions for the *amoA* trees) by inferring the best-fit substitution model using ModelFinder [28] and estimating bootstrap supports using the SH-aLRT test [29]. Trees were visualised in FigTree (http://tree.bio.ed.ac.uk/software/figtree/) and phylogenetic clusters were defined based on strong bootstrap values (> 80% in most cases). Visual assessment of congruence between the two reference trees (*amoA* vs 16S rRNA gene trees) was performed using phylo.io [51] and cluster correspondence was manually assessed based on individual strain correspondence.

For the AOA ‘454-gapped’ Bayesian phylogenetic tree, the best substitution model per codon position estimated using PartitionFinder [52] was SYM + G and GTR + G for the codon position 1 and 2, respectively, and this partition was used to implement two independent Bayesian relaxed molecular clock phylogenetic analyses in BEAST (Bayesian Evolutionary Analysis Sampling Trees) version 1.8 [53] with 5 10^8^ MCMC, using a Yule speciation prior and an uncorrelated lognormal relaxed clock model. Convergence of the two runs was confirmed using Tracer version 1.5 (tree.bio.ed.ac.uk/software/tracer/) and maximum clade credibility trees from converged MCMC runs were generated using TreeAnnotator version 1.7 [53] after 50% of the MCMC steps were removed. Both AOA 454 and AOA 454-gapped tree were compared using phylo.io [51] and treecompare in the DendroPy library [44].

### AOB putative environmental specialisation

Environmental factors (pH, nitrogen and carbon contents, C:N ratio, organic matter content (LOI), moisture content and vegetation type) associated with AOB *amoA* sequence composition were identified by canonical correspondence analysis followed by permutation tests performed on relative abundance matrices using the vegan package [43] in R. Relative abundance matrices were built by blasting each AOB *amoA* sample sequence obtained from the 50 soils against the bacterial non-redundant database clustered at different cut-offs (usearch id 0.9, 0.95, 0.97 and 1), as performed for the archaeal dataset [54]. As pH appeared to be the most significant factor for niche specialisation in this AOB dataset, relative abundance of sequences in the CEH and Craibstone soil samples affiliating within each phylogenetic cluster, based on the MiSeq ‘assembly’ pipeline sequencing technology, were represented using a heatmap.

## Additional file

Additional file 1: **Figure S1.** Full *amoA* (A) and 16S rRNA (B) gene trees for the bacterial ammonia oxidiser reference sequences. **Figure S2.** Congruence of *amoA* and 16S rRNA gene phylogenetic trees for the bacterial ammonia oxidiser reference sequences. Shades of blue indicate similarity between the most common nodes between the two trees. **Figure S3.** Full bacterial *amoA* gene tree including the environmental sequences (assembled using the MiSeq ‘Assembly’ pipeline) and the reference sequences. **Figure S4.** Congruence between two phylogenetic trees of 370 archaeal *amoA* sequences (see Gubry-Rangin et al., 2015) with (A) or without (B) the sequence gap corresponding to the MiSeq AOA gap pipeline. Branch colour corresponds to congruence between the two trees. **Table S1.** Identification of sequences of the 56 terrestrial *Nitrosospira* AOB strains used in this study. For strains with > 1 copy, only the sequences used in this study presented. n.a. – not applicable. **Table S2.** Statistical results of the canonical correspondence and permutation analyses performed on the AOB *amoA* communities clustered at different identity cut-offs. **Table S3.** Number of sequences and richness of AOA and AOB *amoA* sequences retrieved in each Craibstone soil sample with different sequencing technologies, with or without rarefaction to the smallest number of sequences obtained in one of the two technologies. **Table S4.** Characteristics of the 33 UK soils (26 CEH followed by 7 Craibstone soils) used in the multivariate statistics analysis. (DOCX 1560 kb)

## Data Availability

Scripts developed in this work can be found on GitHub (https://github.com/AigleAxel/amoA_MiSeq_sequencing/). Read data have been submitted to the Sequence Read Archive (SRA) under the accession number PRJNA548755.
