# Supplementary material for: The application of high-throughput sequencing technology to analysis of amoA phylogeny and environmental niche specialisation of terrestrial bacterial ammonia-oxidisers
Source: Environ Microbiome. 2019 Jul 4;14:3. doi: 10.1186/s40793-019-0342-6 (PMC7989807; doi:10.1186/s40793-019-0342-6)
Supplement: Supplementary file 1 — Figure S1. Full amoA (A) and 16S rRNA (B) gene trees for the bacterial ammonia oxidiser reference sequences. Figure S2. Congruence of amoA and 16S rRNA gene phylogenetic trees for the bacterial ammonia oxidiser reference sequences. Shades of blue indicate similarity between the most common nodes between the two trees. Figure S3. Full bacterial amoA gene tree including the environmental sequences (assembled using the MiSeq ‘Assembly’ pipeline) and the reference sequences. Figure S4. Congruence between two phylogenetic trees of 370 archaeal amoA sequences (see Gubry-Rangin et al., 2015) with (A) or without (B) the sequence gap corresponding to the MiSeq AOA gap pipeline. Branch colour corresponds to congruence between the two trees. Table S1. Identification of sequences of the 56 terrestrial Nitrosospira AOB strains used in this study. For strains with > 1 copy, only the sequences used in this study presented. n.a. – not applicable. Table S2. Statistical results of the canonical correspondence and permutation analyses performed on the AOB amoA communities clustered at different identity cut-offs. Table S3. Number of sequences and richness of AOA and AOB amoA sequences retrieved in each Craibstone soil sample with different sequencing technologies, with or without rarefaction to the smallest number of sequences obtained in one of the two technologies. Table S4. Characteristics of the 33 UK soils (26 CEH followed by 7 Craibstone soils) used in the multivariate statistics analysis. (DOCX 1560 kb) [file 40793_2019_342_MOESM1_ESM.docx]

**Supplementary Information:**

**Supplementary figures:**

**Figure S1.** Full *amoA* (A) and 16S rRNA (B) gene trees for the bacterial ammonia oxidiser reference sequences.

**A)**

**
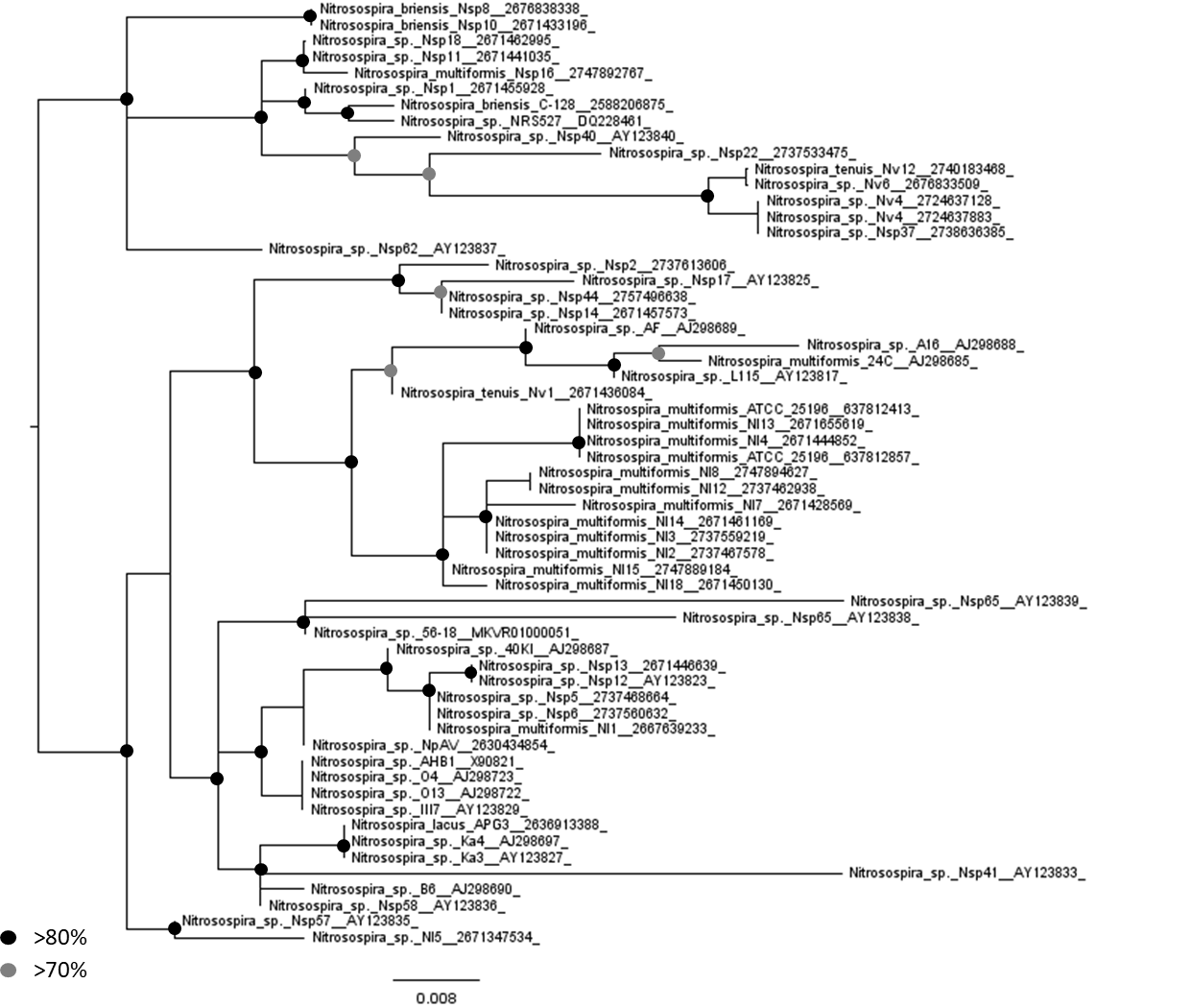
**

**B)**

**
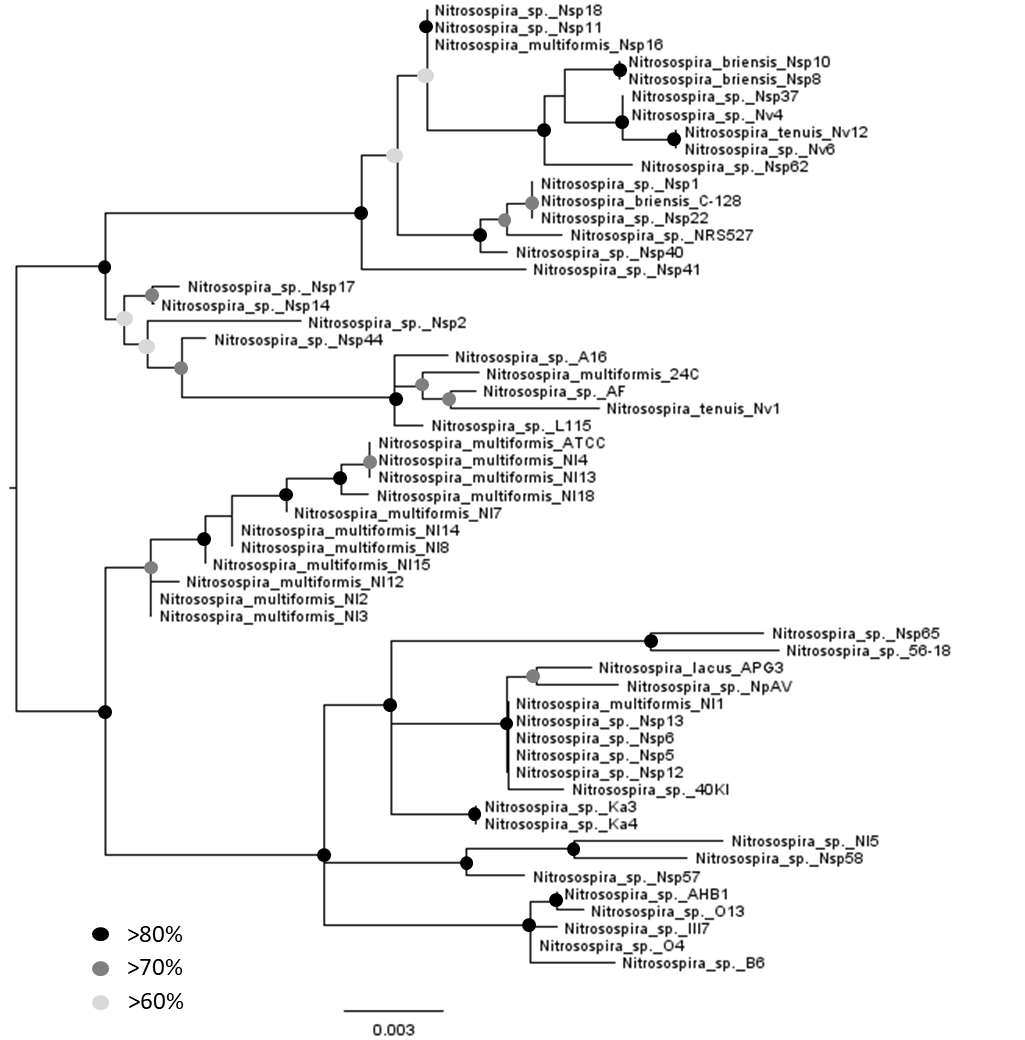
**

**Figure S2.** Congruence of *amoA* and 16S rRNA gene phylogenetic trees for the bacterial ammonia oxidiser reference sequences. Shades of blue indicate similarity between the most common nodes between the two trees.


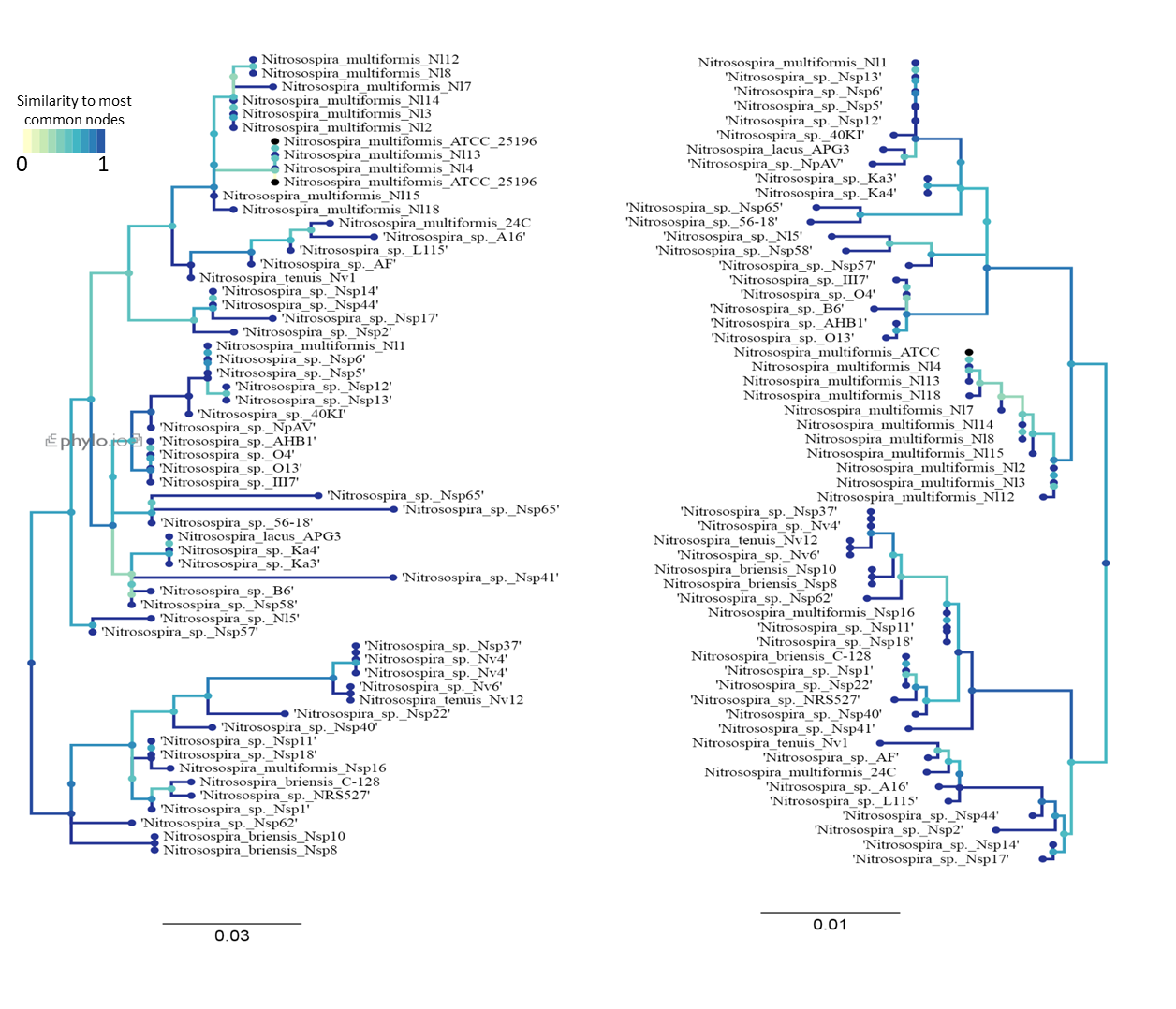


**Figure S3:** Full bacterial *amoA* gene tree including the environmental sequences (assembled using the MiSeq ‘Assembly’ pipeline) and the reference sequences.

**
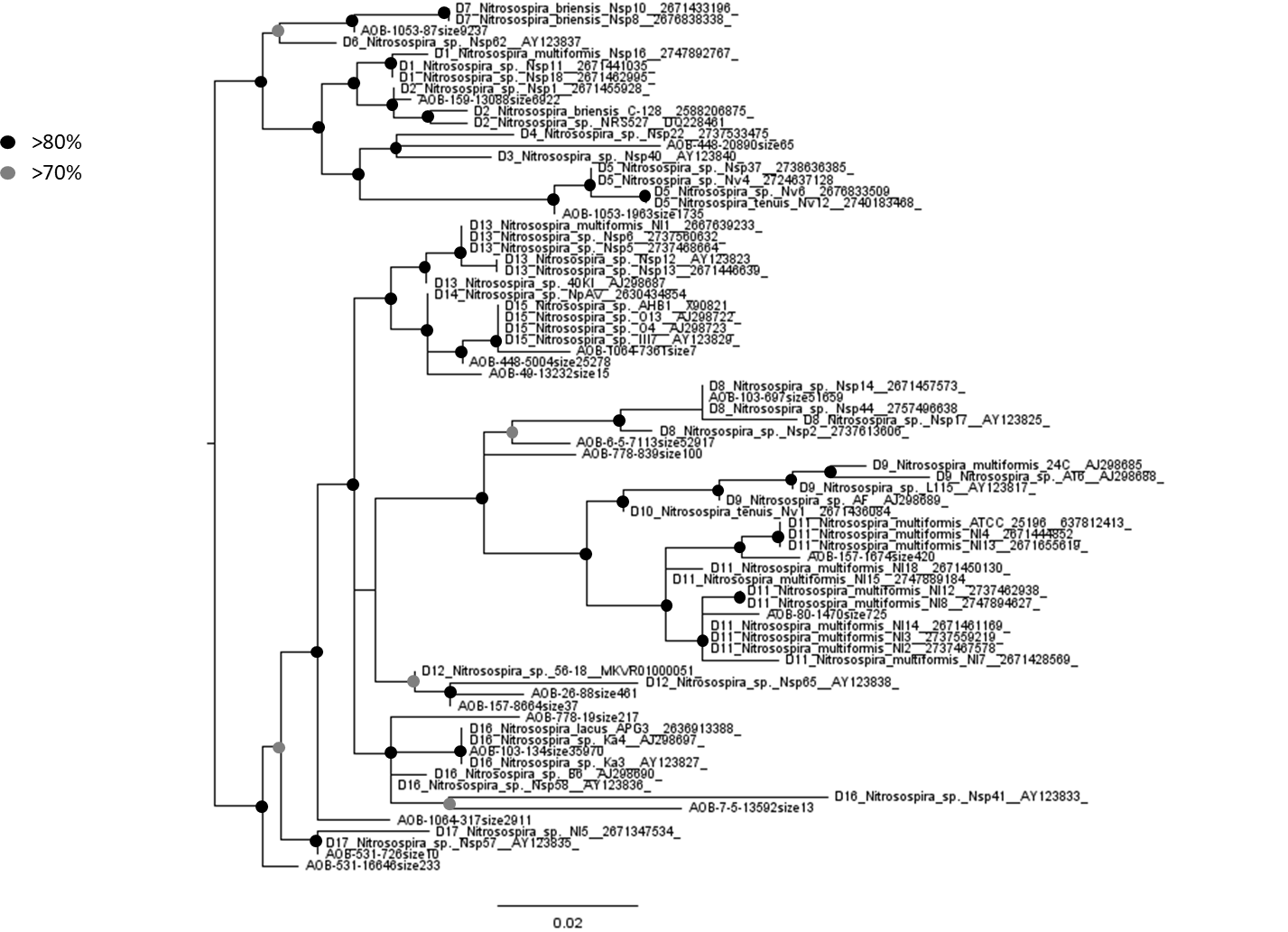
**

**Figure S4:** Congruence between two phylogenetic trees of 370 archaeal *amoA* sequences (see Gubry-Rangin et al., 2015) with (A) or without (B) the sequence gap corresponding to the MiSeq AOA gap pipeline. Branch colour corresponds to congruence between the two trees.

**
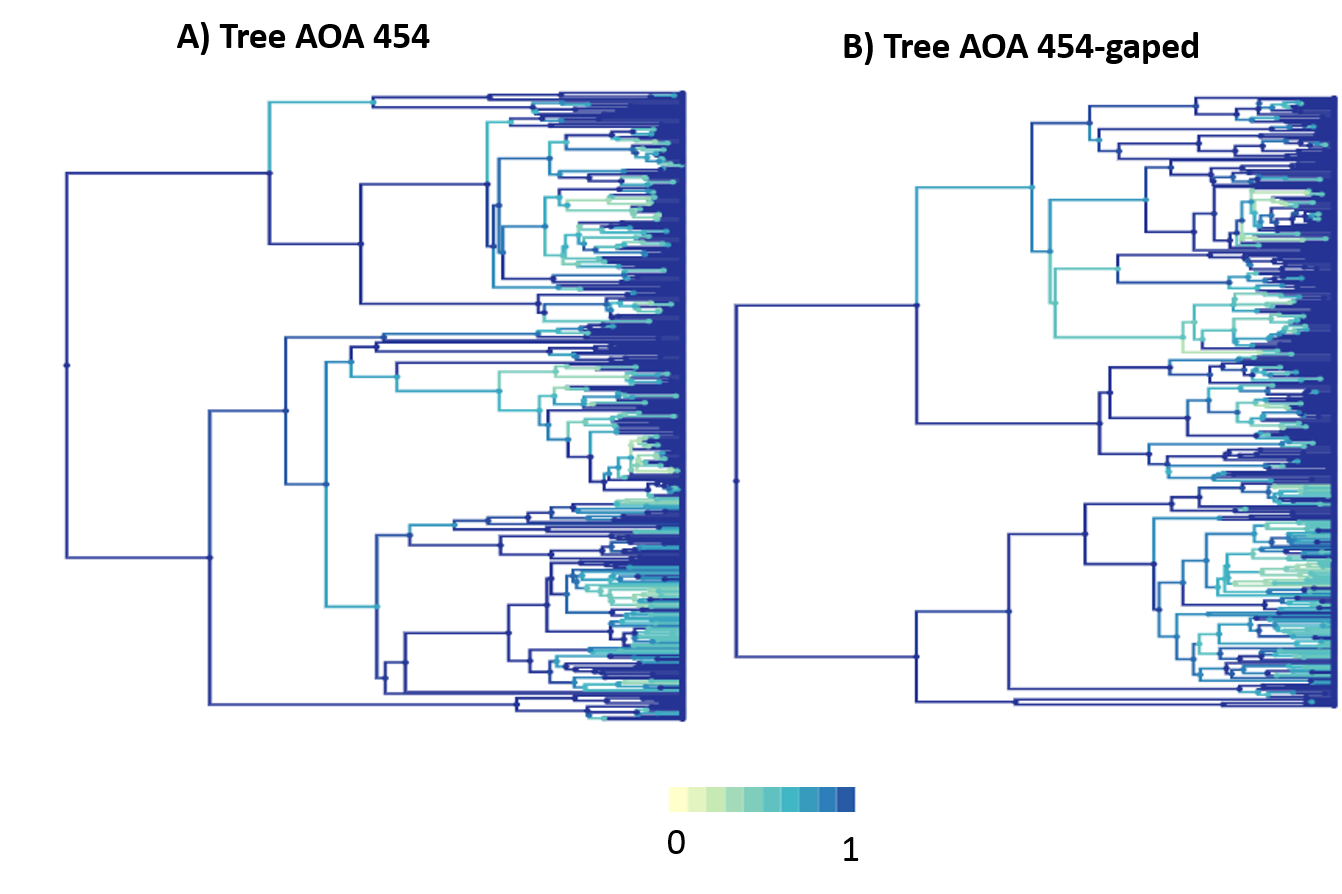
**

**Supplementary tables:**

**Table S1.** Identification of sequences of the 56 terrestrial *Nitrosospira* AOB strains used in this study. For strains with >1 copy, only the sequences used in this study are presented. n.a. – not applicable.

| **Organism** | ***amoA* gene ID** | **16S rRNA gene ID** |
| --- | --- | --- |
| Nitrosospira multiformis Nsp16 | 2747892767 | 2747891151 |
| Nitrosospira sp. Nsp18 | 2671462995 | 2671464162 |
| Nitrosospira sp. 1 Nsp11 | 2671441035 | 2671440229 |
| Nitrosospira briensis C-128 | 2588206875, 2588206327, 2588205525 | NZ_CP012371 |
| Nitrosospira sp. Nsp1 | 2671455928 | 2671452938 |
| Nitrosospira sp. NRS527 | DQ228461 | AF353158 |
| Nitrosospira sp. Nsp22 | 2737533475 | 2737532035 |
| Nitrosospira sp. Nsp40 | AY123840 | AY123787 |
| Nitrosospira multiformis 24C | AF042171 | AF408634 |
| Nitrosospira sp. L115 | AY123817 | AY123796 |
| Nitrosospira tenuis Nv1 | 2671436084 | 2671436341 |
| Nitrosospira sp. A16 | AJ298688 | AJ005544 |
| Nitrosospira sp. AF | AJ298689 | X84658 |
| Nitrosospira briensis Nsp10 | 2671433196 | 2671432470 |
| Nitrosospira briensis Nsp8 | 2676838338 | 2676836691 |
| Nitrosospira sp. Nsp62 | AY123837 | AY123790 |
| Nitrosospira tenuis Nv12 | 2740183468 | 2676833655 |
| Nitrosospira sp. Nv6 | 2676833509 | 2740183743 |
| Nitrosospira sp. Nsp37 | 2738636385 | 2738633597 |
| Nitrosospira sp. Nv4 | 2724637883, 2724637128 | 2724637574 |
| Nitrosospira multiformis ATCC 25196 | 637812857, 637812413, 637810885 | AY123807 |
| Nitrosospira multiformis Nl13 | 2671655619 | 2671654789 |
| Nitrosospira multiformis Nl4 | 2671444852 | 2671442926 |
| Nitrosospira multiformis Nl18 | 2671450130 | 2671449885 |
| Nitrosospira multiformis Nl15 | 2747889184 | 2747888193 |
| Nitrosospira multiformis Nl14 | 2671461169 | 2671459434 |
| Nitrosospira multiformis Nl7 | 2671428569 | 2671429534 |
| Nitrosospira multiformis Nl8 | 2747894627 | 2747894303 |
| Nitrosospira multiformis Nl12 | 2737462938 | 2737462066 |
| Nitrosospira multiformis Nl2 | 2737467578 | 2737465391 |
| Nitrosospira multiformis Nl3 | 2737559219 | 2737556968 |
| Nitrosospira sp. Nsp2 | 2737613606 | 2737612607 |
| Nitrosospira sp. Nsp14 | 2671457573 | 2671456229 |
| Nitrosospira sp. Nsp44 | 2757496638, 2757494294, 2757494089 | 2757496171 |
| Nitrosospira sp. Nsp17 | AY123825 | AY123804 |
| Nitrosospira sp. Nsp5 | 2737468664 | 2737469961 |
| Nitrosospira multiformis Nl1 | 2667639233, 2667638847, 2667636517 | 2667636937 |
| Nitrosospira sp. Nsp13 | 2671446639 | 2671447611 |
| Nitrosospira sp. Nsp6 | 2737560632 | 2737561558 |
| Nitrosospira sp. Nsp12 | AY123823 | AY123801 |
| Nitrosospira sp. 40KI | AJ298687 | X84656 |
| Nitrosospira sp. NpAV | 2630434854 | 2630432220 |
| Nitrosospira sp. Nsp65 | AY123838, AY123839 | AY123813 |
| Nitrosospira sp. 56-18 | MKVR01000051 | MKVR01000014.1:c34397-32855 |
| Nitrosospira sp. III7 | AY123829 | AY123809 |
| Nitrosospira sp. O13 | AJ298722 | AJ012108 |
| Nitrosospira sp. O4 | AJ298723 | AJ012107 |
| Nitrosospira sp. AHB1 | X90821 | X90820 |
| Nitrosospira sp. Ka4 | AJ298697 | AJ012106 |
| Nitrosospira sp. Ka3 | AY123827 | AY123806 |
| Nitrosospira lacus APG3 | 2636913388 | 2636911585 |
| Nitrosospira sp. Nsp41 | AY123833 | AY123788 |
| Nitrosospira sp. B6 | AJ298690 | X84657 |
| Nitrosospira sp. Nsp58 | AY123836 | AY123789 |
| Nitrosospira sp. Nsp57 | AY123835 | AY123791 |
| Nitrosospira sp. Nl5 | 2671347534 | 2671347268 |

**Table S2.** Statistical results of the canonical correspondence and permutation analyses performed on the AOB *amoA* communities clustered at different identity cut-offs.

| **Identity = 100% ; Number of clusters = 3868** |
| --- |
| Call: cca(formula = Clusters ~ pH + C + N + CN + Moisture + LOI +  vegetation, data = Facteur[1:8])  Inertia Proportion Rank  Total 12.6945 1.0000  Constrained 4.5538 0.3587 9  Unconstrained 8.1406 0.6413 23  Inertia is scaled Chi-square  Eigenvalues for constrained axes:  CCA1 CCA2 CCA3 CCA4 CCA5 CCA6 CCA7 CCA8 CCA9  0.8032 0.7069 0.6536 0.5810 0.5085 0.4061 0.3528 0.2890 0.2528  Eigenvalues for unconstrained axes:  CA1 CA2 CA3 CA4 CA5 CA6 CA7 CA8  0.7089 0.6253 0.5917 0.5382 0.4919 0.4831 0.4576 0.4336  (Showing 8 of 23 unconstrained eigenvalues) |
| Permutation test for cca under reduced model  Permutation: free  Number of permutations: 999  Model: cca(formula = Clusters ~ pH + C + N + CN + Moisture + LOI + vegetation, data = Facteur[1:8])  Df ChiSquare F Pr(>F)  Model 9 4.5538 1.4296 0.001 ***  Residual 23 8.1406  ---  Signif. codes: 0 ‘***’ 0.001 ‘**’ 0.01 ‘*’ 0.05 ‘.’ 0.1 ‘ ’ 1 |
| Permutation test for cca under reduced model  Terms added sequentially (first to last)  Permutation: free  Number of permutations: 999  Model: cca(formula = Clusters ~ pH + C + N + CN + Moisture + LOI + vegetation, data = Facteur[1:8])  Df ChiSquare F Pr(>F)  pH 1 0.6672 1.8852 0.001 ***  C 1 0.6593 1.8628 0.001 ***  N 1 0.5577 1.5756 0.001 ***  CN 1 0.5227 1.4769 0.002 **  Moisture 1 0.4820 1.3618 0.015 *  LOI 1 0.4414 1.2472 0.072 .  vegetation 3 1.2235 1.1522 0.106  Residual 23 8.1406  ---  Signif. codes: 0 ‘***’ 0.001 ‘**’ 0.01 ‘*’ 0.05 ‘.’ 0.1 ‘ ’ 1 |
| **Identity = 97% ; Number of clusters = 187** |
| Call: cca(formula = Clusters ~ pH + C + N + CN + Moisture + LOI +  vegetation, data = Facteur[1:8])  Inertia Proportion Rank  Total 4.4355 1.0000  Constrained 2.0083 0.4528 9  Unconstrained 2.4272 0.5472 23  Inertia is scaled Chi-square  Eigenvalues for constrained axes:  CCA1 CCA2 CCA3 CCA4 CCA5 CCA6 CCA7 CCA8 CCA9  0.6766 0.4015 0.3666 0.1763 0.1309 0.1100 0.0694 0.0528 0.0242  Eigenvalues for unconstrained axes:  CA1 CA2 CA3 CA4 CA5 CA6 CA7 CA8  0.4459 0.3753 0.2368 0.1910 0.1812 0.1564 0.1431 0.1231  (Showing 8 of 23 unconstrained eigenvalues) |
| Permutation test for cca under reduced model  Permutation: free  Number of permutations: 999  Model: cca(formula = Clusters ~ pH + C + N + CN + Moisture + LOI + vegetation, data = Facteur[1:8])  Df ChiSquare F Pr(>F)  Model 9 2.0083 2.1146 0.001 ***  Residual 23 2.4272  ---  Signif. codes: 0 ‘***’ 0.001 ‘**’ 0.01 ‘*’ 0.05 ‘.’ 0.1 ‘ ’ 1 |
| Permutation test for cca under reduced model  Terms added sequentially (first to last)  Permutation: free  Number of permutations: 999  Model: cca(formula = Clusters ~ pH + C + N + CN + Moisture + LOI + vegetation, data = Facteur[1:8])  Df ChiSquare F Pr(>F)  pH 1 0.44840 4.2490 0.001 ***  C 1 0.27747 2.6293 0.006 **  N 1 0.20377 1.9309 0.020 *  CN 1 0.28338 2.6853 0.003 **  Moisture 1 0.15234 1.4436 0.112  LOI 1 0.19678 1.8647 0.032 *  vegetation 3 0.44619 1.4094 0.083 .  Residual 23 2.42718  ---  Signif. codes: 0 ‘***’ 0.001 ‘**’ 0.01 ‘*’ 0.05 ‘.’ 0.1 ‘ ’ 1 |
| **Identity = 95% ; Number of clusters = 71** |
| Call: cca(formula = Clusters ~ pH + C + N + CN + Moisture + LOI +  vegetation, data = Facteur[1:8])  Inertia Proportion Rank  Total 3.2686 1.0000  Constrained 1.5383 0.4706 9  Unconstrained 1.7303 0.5294 23  Inertia is scaled Chi-square  Eigenvalues for constrained axes:  CCA1 CCA2 CCA3 CCA4 CCA5 CCA6 CCA7 CCA8 CCA9  0.6433 0.3564 0.1501 0.1477 0.0856 0.0686 0.0377 0.0362 0.0128  Eigenvalues for unconstrained axes:  CA1 CA2 CA3 CA4 CA5 CA6 CA7 CA8  0.3975 0.3461 0.2169 0.1526 0.1356 0.0991 0.0843 0.0800  (Showing 8 of 23 unconstrained eigenvalues) |
| Permutation test for cca under reduced model  Permutation: free  Number of permutations: 999  Model: cca(formula = Clusters ~ pH + C + N + CN + Moisture + LOI + vegetation, data = Facteur[1:8])  Df ChiSquare F Pr(>F)  Model 9 1.5383 2.2719 0.001 ***  Residual 23 1.7303  ---  Signif. codes: 0 ‘***’ 0.001 ‘**’ 0.01 ‘*’ 0.05 ‘.’ 0.1 ‘ ’ 1 |
| Permutation test for cca under reduced model  Terms added sequentially (first to last)  Permutation: free  Number of permutations: 999  Model: cca(formula = Clusters ~ pH + C + N + CN + Moisture + LOI + vegetation, data = Facteur[1:8])  Df ChiSquare F Pr(>F)  pH 1 0.42334 5.6271 0.001 ***  C 1 0.22256 2.9583 0.003 **  N 1 0.17875 2.3759 0.016 *  CN 1 0.23958 3.1845 0.002 **  Moisture 1 0.09317 1.2385 0.264  LOI 1 0.10060 1.3372 0.198  vegetation 3 0.28028 1.2418 0.172  Residual 23 1.73034  ---  Signif. codes: 0 ‘***’ 0.001 ‘**’ 0.01 ‘*’ 0.05 ‘.’ 0.1 ‘ ’ 1 |
| **Identity = 90% ; Number of clusters = 14** |
| Call: cca(formula = Clusters ~ pH + C + N + CN + Moisture + LOI +  vegetation, data = Facteur[1:8])  Inertia Proportion Rank  Total 1.4662 1.0000  Constrained 0.7205 0.4914 9  Unconstrained 0.7457 0.5086 12  Inertia is scaled Chi-square  Eigenvalues for constrained axes:  CCA1 CCA2 CCA3 CCA4 CCA5 CCA6 CCA7 CCA8 CCA9  0.4842 0.1066 0.0653 0.0241 0.0192 0.0187 0.0018 0.0005 0.0001  Eigenvalues for unconstrained axes:  CA1 CA2 CA3 CA4 CA5 CA6 CA7 CA8 CA9 CA10  0.31029 0.19680 0.11418 0.05716 0.03395 0.01682 0.01435 0.00089 0.00047 0.00046  CA11 CA12  0.00023 0.00012 |
| Permutation test for cca under reduced model  Permutation: free  Number of permutations: 999  Model: cca(formula = Clusters ~ pH + C + N + CN + Moisture + LOI + vegetation, data = Facteur[1:8])  Df ChiSquare F Pr(>F)  Model 9 0.72051 2.4692 0.001 ***  Residual 23 0.74572  ---  Signif. codes: 0 ‘***’ 0.001 ‘**’ 0.01 ‘*’ 0.05 ‘.’ 0.1 ‘ ’ 1 |
| Permutation test for cca under reduced model  Terms added sequentially (first to last)  Permutation: free  Number of permutations: 999  Model: cca(formula = Clusters ~ pH + C + N + CN + Moisture + LOI + vegetation, data = Facteur[1:8])  Df ChiSquare F Pr(>F)  pH 1 0.25949 8.0034 0.001 ***  C 1 0.04258 1.3132 0.274  N 1 0.05290 1.6316 0.166  CN 1 0.17623 5.4355 0.001 ***  Moisture 1 0.02816 0.8685 0.459  LOI 1 0.05754 1.7746 0.139  vegetation 3 0.10361 1.0652 0.406  Residual 23 0.74572  ---  Signif. codes: 0 ‘***’ 0.001 ‘**’ 0.01 ‘*’ 0.05 ‘.’ 0.1 ‘ ’ 1 |

**Table S3.** Number of sequences and richness of AOA and AOB *amoA* sequences retrieved in each Craibstone soil sample with different sequencing technologies, with or without rarefaction to the smallest number of sequences obtained in one of the two technologies.

| **Organisms** | **Technologies** | **Total # reads** | | | **OTU richness** | | | | | | | | |
| --- | --- | --- | --- | --- | --- | --- | --- | --- | --- | --- | --- | --- | --- |
|  |  | **Raw reads** | **Post filtering** | **Post cleaning/assembly** | **Rare-faction** | **# sequences** | **4.5** | **5** | **5.5** | **6** | **6.5** | **7** | **7.5** |
| **AOA** | 454 | 42,193 | N/A | 9,429 (2,474 clusters) | N/A | 868 | 233 | 328 | 174 | 147 | 261 | 331 | 354 |
|  | MiSeq “gap” | 358,872 | 260,413 | 74,771 (775 clusters) | Without | 8,287 | 194 | 230 | 295 | 139 | 114 | 184 | 153 |
|  |  |  |  |  | With | 868 | 107 | 162 | 189 | 113 | 130 | 204 | 165 |
| **AOB** | MiSeq “assembly” | 377,172 | 204,315 | 40,509 (902 clusters) | N/A | 4,261 | 61 | 283 | 203 | 220 | 224 | 212 | 209 |
|  | MiSeq “gap” | 377,172 | 258,688 | 75,349 (1,016 clusters) | Without | 8,160 | 68 | 347 | 267 | 224 | 247 | 252 | 253 |
|  |  |  |  |  | With | 4,261 | 67 | 317 | 248 | 217 | 233 | 227 | 236 |

**Table S4.** Characteristics of the 33 UK soils (26 CEH followed by 7 Craibstone soils) used in the multivariate statistics analysis.

| **Site** | **pH** | **C (%)** | **N (%)** | **C:N ratio** | **H_2_O (%)** | **Organic matter (%)** | **Simplified aggregate vegetation class** |
| --- | --- | --- | --- | --- | --- | --- | --- |
| **1** | 4.4 | 3.3 | 0.39 | 8.46 | 26.7 | 6.58 | grassland |
| **2** | 4.8 | 2.1 | 0.22 | 9.55 | 22.8 | 4.88 | grassland |
| **3** | 7.6 | 3.2 | 0.34 | 9.41 | 30.4 | 6.26 | agricultural |
| **4** | 8.3 | 19 | 1.28 | 14.84 | 47.5 | 24.1 | grassland |
| **5** | 8.5 | 11 | 0.7 | 15.71 | 16.4 | 12.08 | grassland |
| **6** | 6.9 | 2 | 0.16 | 12.5 | 24.1 | 4.14 | grassland |
| **7** | 7.7 | 5.3 | 0.56 | 9.46 | 34 | 11.17 | grassland |
| **8** | 6.7 | 1.4 | 0.13 | 10.77 | 15.4 | 2.91 | agricultural |
| **9** | 8.1 | 2.9 | 0.31 | 9.35 | 23.3 | 5.82 | agricultural |
| **10** | 7.9 | 1.9 | 0.18 | 10.56 | 17.7 | 4.03 | forest |
| **11** | 8.7 | 1.3 | 0.13 | 10 | 15.8 | 2.66 | agricultural |
| **12** | 5.3 | 2.1 | 0.2 | 10.5 | 14.5 | 5 | grassland |
| **13** | 6.4 | 1.5 | 0.15 | 9.63 | 35.2 | 10.05 | grassland |
| **14** | 8.2 | 1.7 | 0.18 | 9.44 | 19.9 | 3.72 | grassland |
| **15** | 6.2 | 3.1 | 0.31 | 10 | 29.2 | 5.78 | grassland |
| **16** | 6.8 | 13 | 0.7 | 18.57 | 55.9 | 20.1 | forest |
| **17** | 6.8 | 7.5 | 0.51 | 14.71 | 35.6 | 12.29 | grassland |
| **18** | 8.1 | 2.5 | 0.23 | 10.87 | 20.6 | 5.1 | agricultural |
| **19** | 8.6 | 5.4 | 0.35 | 15.43 | 27.1 | 6.23 | agricultural |
| **20** | 7.2 | 2.5 | 0.17 | 14.71 | 15.8 | 4.51 | agricultural |
| **21** | 6.4 | 3.4 | 0.21 | 16.19 | 14.2 | 5.29 | agricultural |
| **22** | 3.5 | 22.6 | 1.08 | 20.93 | 75.2 | 40.63 | moorland |
| **23** | 6.9 | 1.7 | 0.16 | 10.63 | 15.7 | 3.2 | grassland |
| **24** | 5.6 | 8 | 0.57 | 14.04 | 46.3 | 15.99 | grassland |
| **25** | 8.5 | 4.5 | 0.31 | 14.52 | 33 | 8.91 | grassland |
| **26** | 6.7 | 4.7 | 0.41 | 11.46 | 38.4 | 10 | agricultural |
| **27** | 4.5 | 7.02 | 0.38 | 18.64 | 29.7 | 12.07 | agricultural |
| **28** | 5 | 6.6 | 0.37 | 18.03 | 30.9 | 11.35 | agricultural |
| **29** | 5.5 | 7.42 | 0.4 | 18.45 | 31 | 12.77 | agricultural |
| **30** | 6 | 6.57 | 0.31 | 21.43 | 30.2 | 11.29 | agricultural |
| **31** | 6.5 | 6.42 | 0.29 | 22.03 | 29.4 | 11.04 | agricultural |
| **32** | 7 | 7.97 | 0.36 | 22.09 | 31 | 13.71 | agricultural |
| **33** | 7.5 | 7.14 | 0.34 | 20.84 | 29.1 | 12.28 | agricultural |
